# Wi-Fi CSI-Based Outdoor Human Flow Prediction Using a Support Vector Machine †

**DOI:** 10.3390/s20072141

**Published:** 2020-04-10

**Authors:** Masakatsu Ogawa, Hirofumi Munetomo

**Affiliations:** Faculty of Science and Technology, Sophia University, Tokyo 102-8554, Japan; h-munetomo-4ws@eagle.sophia.ac.jp

**Keywords:** Wi-Fi, channel state information, human flow, machine learning, support vector machine

## Abstract

This paper proposes a channel state information (CSI)-based prediction method of a human flow that includes activity. The objective of the paper is to predict a human flow in an outdoor road. This human flow prediction is useful for the prediction of the number of passing people and their activity without privacy issues as a result of the absence of any camera systems. In this paper, we assume seven types of activities: one, two, and three people walking; one, two, and three people running; and one person cycling. Since the CSI can effectively express the effect of multipath fading in wireless signals, we expected the CSI to predict the various activities. In our proposed method, the amplitude and phase components are extracted from the measured CSI. The feature values for machine learning are the mean and variance of the maximum eigenvalue derived from the auto-correlation matrix and variance–covariance matrix composed of the amplitude or phase components and the passing time of flow. Using these feature values, we evaluated the prediction accuracy by leave-one-out cross-validation with a linear support vector machine (SVM). As a result, the proposed method achieved the maximum prediction accuracy of 100% for each direction and 99.5% for two directions.

## 1. Introduction

Recently, expectations for sensing methods without any cameras have increased. In September 2019, the IEEE802.11 Committee established the project of channel state information (CSI)-based sensing. As of March 2020, the project name is SENS TIG [1]. The advantage of CSI-based sensing compared with camera-based sensing is that it addresses privacy issues. As of March 2020, to the best of our knowledge, there is only one current commercial service. This service, which started in July 2019, is the human presence detection at a shower room in Narita airport, Japan [2]. The camera cannot be installed in the shower room because of privacy issues. Thus, the service is required to assure privacy. This service is one of the use cases for CSI-based sensing.

There are alternative methods of sensing, such as the use of a passive infrared ray (PIR) sensor or light detection and ranging (LiDAR) sensor. PIR sensors cover a low range, require multiple sensors to achieve reliable detection, and the detection accuracy is low for static persons [3]. For example, three PIR sensors are used to detect people’s movement [4]. On the other hand, LiDAR sensors are a crucial component for autonomous cars, but LiDAR sensors are not cheaper than Wi-Fi devices [5]. We expect Wi-Fi devices with CSI collection to be a cost-effective solution. Once the standardization is completed, CSI collection capability will be incorporated into Wi-Fi devices. The production volume of Wi-Fi devices is enormous, so we expect a price decline.

The IEEE802.11n/ac/ax standard applies the orthogonal frequency division multiplex (OFDM) and multiple input and multiple output (MIMO) technologies. The OFDM is a multi-carrier transmission using multiple subcarriers concerning the physical layer information. Since the subcarrier characteristic is different because of the effect of multipath propagation, the characteristic varies with the location and shape of objects. Thus, the information about the frequency domain is obtained by OFDM transmission. MIMO uses multiple antennas in the transmitter and receiver. Since the antenna location is different for each antenna, the effect of multipath propagation is different for each transmitting and receiving antenna pair. Thus, the information about the space domain is obtained by MIMO transmission. The CSI consists of frequency and space information, expresses the effect of multipath propagation, and corresponds to the space state between transmitting and receiving antennas. To date, CSI has been used for various studies such as human movement detection, human behavior classification, location estimation, object identification, human counting in a room, and human counting in a passageway [3,5,6,7,8,9,10,11,12,13,14,15,16,17,18,19].

**Figure 1:**
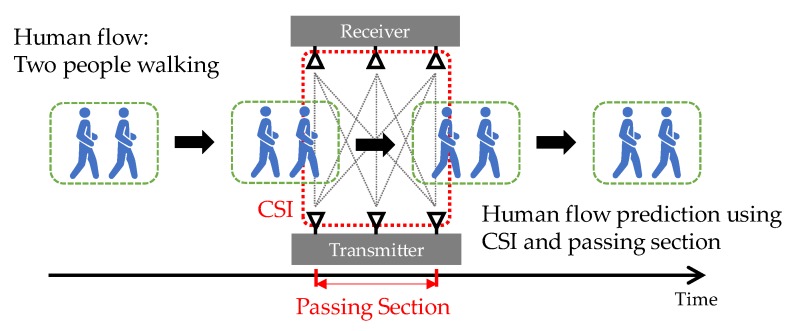
Overview of human flow prediction.

The objective of the paper is a prediction of human flow including various activities: one, two, and three people walking; one, two, and three people running; and one person cycling. In the example shown in Figure 1, the human activity is two people walking. At first, to detect the human flow, the fluctuation of CSI is used. The CSI expresses the space state between transmitting and receiving antennas. If an object moves around between antennas, the fluctuation of CSI is larger than no object. The fluctuation in CSI while a person is passing depends on the activity. When running is compared with walking, the fluctuation of the CSI for running is generally larger. Thus, the CSI is useful for detecting human flow and classifying the number of people and their activities. When people walk, keeping a certain distance from the person in front, the length of the passage section, i.e., passing time, between antennas depends on the number of people within the human flow and also depends on the activity. Thus, the length of the passage section is also useful for classifying the number of people and their activities. To improve the classification accuracy, the antenna spacing was set to be large, 1.5 m, in the transmitter and receiver. This is an innovative idea, and the scanning area for the CSI becomes more extensive as the antenna spacing is physically increased. The antennas in the transmitter and receiver were installed diagonally to the passing direction to expand the scanning area further. Our idea is similar to that of [16]. This reference focuses on the classification of vehicles. The study used the transmitting antennas on one side of the outdoor road and the receiving antennas on the other side of the road. The experimental result described that, comparing the case where the vehicle passes close to the transmitting antennas, when the vehicle passes close to the receiving antennas, the space of each transmitting and receiving antenna pair widens, and radio waves cover the vehicle body efficiently. It can be interpreted that the correlation of each transmitting and receiving antenna pair decreases when it is away from the transmitting antenna. This idea corresponds to increasing the antenna spacing, that is, our idea.

In this study, a human flow that includes activity was classified by a support vector machine (SVM), and we clarify the feature values to achieve the best accuracy for the classification.

This paper is the extended version of the conference paper [20]. We conducted additional evaluations, and the detailed explanation has been added and revised drastically. The paper is organized as follows: Section 2 reviews related studies concerning the prediction system using the CSI. Then, Section 3 describes the mathematical expression of CSI. Section 4 presents the proposed system for human flow prediction. Section 5 details experiments on the proposed system and evaluates the prediction accuracy. Section 6 discusses the comparison with the conventional method. Section 7 concludes this paper and points out future studies.

## 2. Related Studies

CSI can effectively express the effect of multipath propagation and multipath fading in wireless signals; thus, CSI-based estimation is suitable for environments where diffraction and scattering occur. Such situations are indoor, and many studies have been conducted indoors rather than outdoors. To date, CSI has been used for various studies such as human movement detection, human behavior classification, location estimation, object identification, human counting in a room, and human counting in a passageway [3,5,6,7,8,9,10,11,12,13,14,15,16,17,18,19].

The related studies can be classified into non-moving and moving objects. In the case of non-moving objects, the multipath propagation is different depending on the location or the shape of objects where they exist between antennas. This characteristic is used for location estimation and object identification [6,7,8,9,10] among others. In these applications, the main classification algorithms for estimation, such as SVMs and convolutional neural networks (CNNs), are used. In the case of moving objects, the fluctuation of multipath fading is different depending on human activity and/or the number of moving people. This characteristic is used for human movement detection, human behavior classification, human or vehicle identification, human counting in a room, and human counting in a passageway [3,5,11,12,13,14,15,16,17,18,19], among others. In these applications, the main classification algorithms for estimation, such as SVMs, recurrent neural networks (RNNs), and long short-term memory (LSTM), are used. The difference between an SVM and deep learning is that deep learning tools such as CNNs, RNNs, and LSTM generally require much computation time. Considering the computation time, we applied an SVM.

Since the objective of the paper is a prediction of human flow, our proposed system was categorized into moving objects. Qian, K. et al. proposed the human detection method concerning human movement in an indoor passageway [11]. This method uses the feature values derived from the maximum eigenvalue of the variance–covariance matrix composed of CSI and classifies the type of human movements as stationary or moving. Our proposed system borrows this idea, but there is a problem in that the maximum eigenvalue of the variance–covariance matrix is difficult to be reflected in the characteristic when people are passing.

## 3. Channel State Information (CSI)

Current Wi-Fi systems, such as IEEE802.11n/ac/ax, have functions of the OFDM and MIMO. Since the OFDM transmission uses multiple subcarriers, the effects of diffraction and scattering in the wireless signal are reflected in each subcarrier fluctuation. MIMO transmission makes multiple transmission paths composed of transmission and reception antenna pairs, and the CSI is different for each path. In Linux 802.11n CSI Tool, the CSI is obtained whenever the receiver receives the frame [21]. Let *Tx* and *Rx* be transmitting and receiving antenna numbers, respectively, and let *N_Tx_* and *N_Rx_* be the number of transmitting and receiving antennas, respectively. The CSI matrix can be expressed as follows:

(1) $$H_{k}=\left[\begin{array}{ccc} h_{11,k} & \cdots & h_{1N_{T_{x}},k}\\ \vdots & h_{R_{x}T_{x},k} & \vdots \\ h_{N_{R_{x}}1,k} & \cdots & h_{N_{R_{x}}N_{T_{x}},k} \end{array}\right],$$

where *k* denotes the received frame number. Linux 802.11n CSI Tool can obtain CSI on 30 subcarriers. Thus, let *n* be a subcarrier number, and each element of the matrix is expressed as

(2) $$h_{R_{x}T_{x},k}=\left[h_{R_{x}T_{x},k,f_{1}},\cdots ,h_{R_{x}T_{x},k,f_{n}},\cdots ,h_{R_{x}T_{x},k,f_{30}}\right].$$

$h_{R_{x}T_{x},k,f_{n}}$ for subcarrier *n* contains the amplitude and phase components and can be expressed as follows:

(3) $$h_{R_{x}T_{x},k,f_{n}}=\left|h_{R_{x}T_{x},k,f_{n}}\right|e^{∠h_{R_{x}T_{x},k,f_{n}}}.$$

The phase in each subcarrier is

(4) $$∠h_{R_{x}T_{x},k}=\left[∠h_{R_{x}T_{x},k,f_{1}},\cdots ,∠h_{R_{x}T_{x},k,f_{n}},\cdots ,∠h_{R_{x}T_{x},k,f_{30}}\right].$$

The measured phase ${\hat{\varphi }}_{n}$ for subcarrier *n* contains the effect of the timing and phase offset at the receiver. To eliminate the effect, this paper applies the following sanitized phase [11,12] instead of the measured phase. The sanitized phase ${\tilde{\varphi }}_{n}$ is expressed as follows:

(5) $${\tilde{\varphi }}_{n}={\hat{\varphi }}_{n}-\frac{{\hat{\varphi }}_{30}-{\hat{\varphi }}_{1}}{f_{30}-f_{1}}-\frac{1}{30}\sum_{n=1}^{30}{\hat{\varphi }}_{n}.$$

## 4. Proposed System

**Figure 2:**
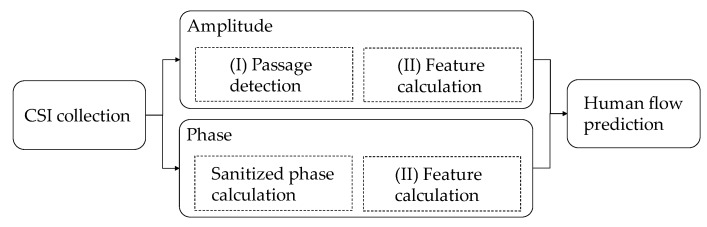
Overview of the proposed system.

The purpose of the proposal is to predict a human flow, including activities, such as one person or two people walking or running, between transmitting and receiving antennas. The proposed system is composed of mainly three parts, as shown in Figure 2: (I) the passage detection of human flow, (II) the calculation of feature values, and (III) the human flow prediction. In the system, the sampling frequency for measuring CSI is set to 1 kHz [12]. In general, if the sampling frequency is low, it is difficult to follow a rapid motion.

### 4.1. Passage Detection of Human Flow

For the passage detection of human flow, the CSI amplitude is used, but the amplitude includes high-frequency noise [14,15]. The reason why the CSI amplitude is used is that the fluctuation of the CSI phase is larger than that of CSI amplitude even if a human flow does not move across transmitting and receiving antennas. To remove the noise, we applied a low pass filter with a cutoff frequency of 10 Hz to the amplitude component [14,15]. Note that the low pass filter is applied to the amplitude in the time domain, not the frequency domain. For confirmation, we tried 1 Hz, 10 Hz, 50 Hz, and 100 Hz filters. When the cutoff frequency was 1Hz, the fluctuation when a human flow moves did not appear adequately due to the low frequency. On the other hand, when the cutoff frequency was 50 Hz or 100 Hz, the fluctuation of value when no human flow moves remained due to the high frequency.

When a human moves across transmitting and receiving antennas, multipath fading occurs and the amplitude fluctuates between received frames. The correlation of amplitude among the frames decreases with the increase of fluctuation. In short, the amplitude characteristics in each subcarrier vary frame-by-frame. Focusing on the relative amplitude between CSI in frames, the amplitude is normalized as follows:

(6) $$\bar{\left|h_{R_{x}T_{x},k}\right|}=\left[\frac{\left|h_{R_{x}T_{x},k,f_{1}}\right|}{\left\|h_{R_{x}T_{x},k}\right\|},\cdots ,\frac{\left|h_{R_{x}T_{x},k,f_{n}}\right|}{\left\|h_{R_{x}T_{x},k}\right\|},\cdots ,\frac{\left|h_{R_{x}T_{x},k,f_{30}}\right|}{\left\|h_{R_{x}T_{x},k}\right\|}\right].$$

Next, we calculated the maximum eigenvalue of the auto-correlation matrix with *window* size using Equation (7). The *window* size corresponds to the number of frames. In the proposed system, the *window* size was set to 10. Let $\alpha_{R_{x}T_{x},k}$ be the maximum eigenvalue of amplitude.

(7) $$\alpha_{R_{x}T_{x},k}=max\left(eig\left(\left[\begin{array}{c} \bar{\left|h_{R_{x}T_{x},k}\right|}\\ \vdots \\ \bar{\left|h_{R_{x}T_{x},k+window-1}\right|} \end{array}\right]{\left[\begin{array}{c} \bar{\left|h_{R_{x}T_{x},k}\right|}\\ \vdots \\ \bar{\left|h_{R_{x}T_{x},k+window-1}\right|} \end{array}\right]}^{T}/window\right)\right).$$

If a human does not move across transmitting and receiving antennas, the correlation is one. The matrix $\left[\cdot \right]{\left[\cdot \right]}^{T}$ is a square matrix of *window* order whose elements are all one. In this case, the maximum eigenvalue in Equation (7) is one.

**Figure 3:**
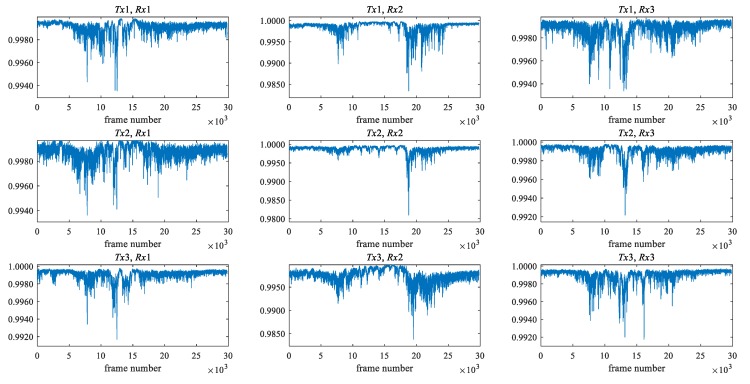
The maximum eigenvalue characteristics of channel state information (CSI) amplitude when the low pass filter is not applied.

**Figure 4:**
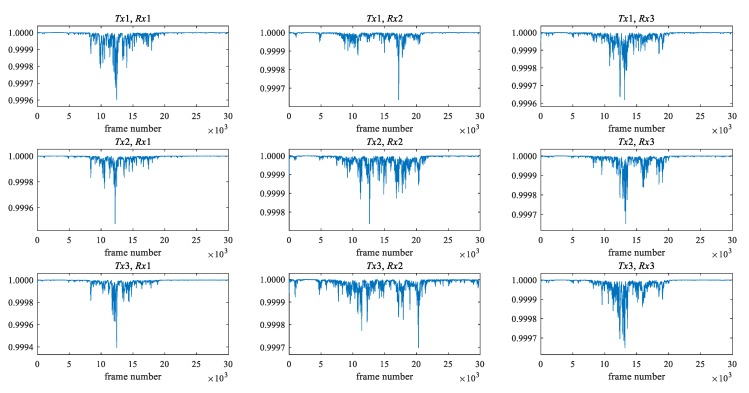
The maximum eigenvalue characteristics of CSI amplitude when the low pass filter is applied.

Figure 3 and Figure 4 show the maximum eigenvalue characteristics of CSI amplitude when the low pass filter is not applied and when the filter is applied, respectively. As shown in Figure 1, two people start walking in front of the antenna, cross it, and pass it. In the environment, there are three transmitting and three receiving antennas, i.e., nine transmission paths. When the filter is not applied, the noise is also included in the maximum eigenvalue characteristics, as shown in Figure 3, but as shown in Figure 4, it is clear that the noise is removed. The eigenvalue is lower than one where two people are passing between antennas.

**Figure 5:**
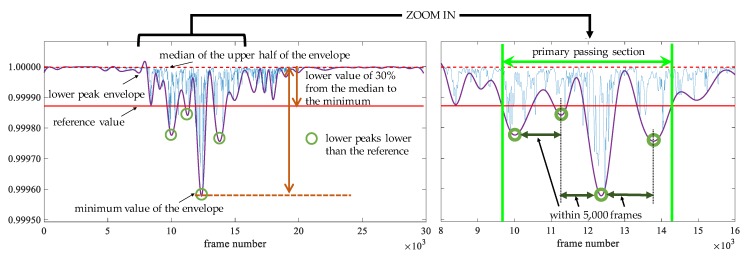
Primary passing section in each path.

**Figure 6:**
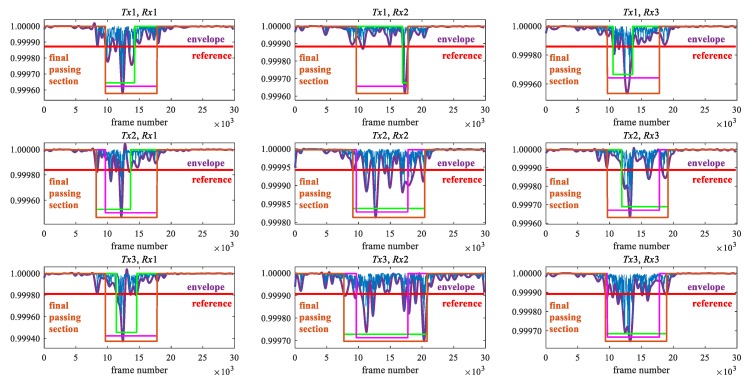
Final passing section in each path.

In the passage detection, we assume that only one human flow is passed during observation. We describe the explanation of the detection method using Figure 5 and Figure 6. Figure 5 shows the *Tx*1–*Rx*1 path in Figure 6. The blue line in Figure 5 is the maximum eigenvalue after the low pass filter, which is the same as Figure 4. Since the fluctuation of the maximum eigenvalue is large while a human flow is passing, we use the lower peak envelope of the maximum eigenvalue smoothed over 300 sample intervals (purple line in Figure 5). The reference value of the envelope (red line in Figure 5) to detect the flow is the lower value of 30% from the median of the upper half of the envelope (red dot line in Figure 5) to the minimum value. Next, the lower peaks, i.e., valley, that are lower than the reference value are selected (green circle in Figure 5). Among the selected lower peaks, the adjacent peaks within 5000 frames are extracted. The section of the lower peak envelope with less than the threshold including extracted lower peak is called a primary passing section (light green line in Figure 5). Finally, the final passing section is determined by comparing the primary passing section in each transmission path with the median values of the start and end points in the primary passing sections of all transmission paths. The final passing section of the start and end points in each transmission path is the minimum to maximum frame number among the primary passing section and the median values. The ranges of green and pink lines in Figure 6 show the primary passing sections in each path and the median of the primary passing section in all paths, respectively. The range of the brown line is the final passing section in each path. The reason why the final passing section is applied is that the primary passing section may not be appropriate depending on the transmission path. For example, the primary passing section in the *Tx*2–*Rx*3 path is very narrow and cannot be appropriately detected. To address this problem, we have to apply the final passing section instead of the primary passing section.

### 4.2. Calculation of Feature Values and Human Flow Prediction.

In this system, the feature values are created from the amplitude and phase components of CSI. The lengths of the final passing section in each transmission path are also used as the feature values. As with the CSI amplitude, the maximum eigenvalue of phase, $\beta_{R_{x}T_{x},k}$, is calculated in Equations (8) and (9).

(8) $$\bar{∠h_{R_{x}T_{x},k}}=\left[\frac{∠h_{R_{x}T_{x},k,f_{1}}}{\left\|∠h_{R_{x}T_{x},k}\right\|},\cdots ,\frac{∠h_{R_{x}T_{x},k,f_{n}}}{\left\|∠h_{R_{x}T_{x},k}\right\|},\cdots ,\frac{∠h_{R_{x}T_{x},k,f_{30}}}{\left\|∠h_{R_{x}T_{x},k}\right\|}\right].$$

(9) $$\beta_{R_{x}T_{x},k}=max\left(eig\left(\left[\begin{array}{c} \bar{∠h_{R_{x}T_{x},k}}\\ \vdots \\ \bar{∠h_{R_{x}T_{x},k+window-1}} \end{array}\right]{\left[\begin{array}{c} \bar{∠h_{R_{x}T_{x},k}}\\ \vdots \\ \bar{∠h_{R_{x}T_{x},k+window-1}} \end{array}\right]}^{T}/window\right)\right).$$

In addition to the two types of eigenvalues, $\alpha_{R_{x}T_{x},k}$ and $\beta_{R_{x}T_{x},k}$, the maximum eigenvalues derived from the variance–covariance matrix composed of the CSI amplitude or the phase CSI phase in Equations (10) and (11) are used. This method to create the maximum eigenvalues is the same as that of [11].

(10) $$\gamma_{R_{x}T_{x},k}=max\left(eig\left(\begin{array}{c} \left[\begin{array}{c} \bar{\left|h_{R_{x}T_{x},k}\right|}-E\left[\bar{\left|h_{R_{x}T_{x},k}\right|}\right]\\ \vdots \\ \bar{\left|h_{R_{x}T_{x},k+window-1}\right|}-E\left[\bar{\left|h_{R_{x}T_{x},k+window-1}\right|}\right] \end{array}\right]\\ {\left[\begin{array}{c} \bar{\left|h_{R_{x}T_{x},k}\right|}-E\left[\bar{\left|h_{R_{x}T_{x},k}\right|}\right]\\ \vdots \\ \bar{\left|h_{R_{x}T_{x},k+window-1}\right|}-E\left[\bar{\left|h_{R_{x}T_{x},k+window-1}\right|}\right] \end{array}\right]}^{T}/window \end{array}\right)\right).$$

(11) $$\delta_{R_{x}T_{x},k}=max\left(eig(\begin{array}{c} {}[\begin{array}{c} \bar{∠h_{R_{x}T_{x},k}}-E\left[\bar{∠h_{R_{x}T_{x},k}}\right]\\ \vdots \\ \bar{∠h_{R_{x}T_{x},k+window-1}}-E\left[\bar{∠h_{R_{x}T_{x},k+window-1}}\right] \end{array}]\\ {\left[\begin{array}{c} \bar{∠h_{R_{x}T_{x},k}}-E\left[\bar{∠h_{R_{x}T_{x},k}}\right]\\ \vdots \\ \bar{∠h_{R_{x}T_{x},k+window-1}}-E\left[\bar{∠h_{R_{x}T_{x},k+window-1}}\right] \end{array}\right]}^{T}/window \end{array})\right).$$

**Figure 7:**
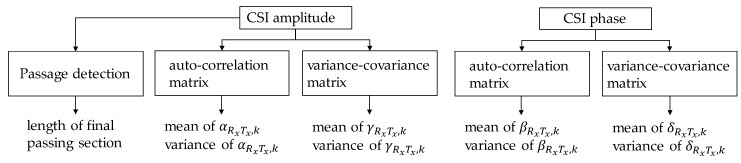
Summary of feature values.

Using the frame number extracted by the final passing sections, the feature values used in the system are the mean and variance of $\alpha_{R_{x}T_{x},k}$, $\beta_{R_{x}T_{x},k}$, $\gamma_{R_{x}T_{x},k}$, and $\delta_{R_{x}T_{x},k}$. Note that the range of *k* is in the final passing section in each transmission path. The lengths of the final passing section in each transmission path are also used as feature values. Specifically, since 3 × 3 MIMO has nine transmission paths, the maximum number of feature values is 4 eigenvalues ($\alpha_{R_{x}T_{x},k}$, $\beta_{R_{x}T_{x},k}$, $\gamma_{R_{x}T_{x},k}$, and $\delta_{R_{x}T_{x},k}$) × 2 (mean and variance) × 9 paths + 1 length of final passing section × 9 paths = 81. The feature values are summarized in Figure 7. The lengths of the final passing section, and the mean and variance of $\alpha_{R_{x}T_{x},k}$ and $\gamma_{R_{x}T_{x},k}$ are derived from the CSI amplitude. The mean and variance of $\beta_{R_{x}T_{x},k}$ and $\delta_{R_{x}T_{x},k}$ are derived from the CSI phase.

When a human flow has passed, at first, the system detects a human flow, and next the system predicts the number of people and activity of human flow using the linear SVM.

## 5. Evaluation

We derived the feature values to detect a human flow and to classify its activity. In this section, we evaluate the proposed system to clarify the useful feature values for achieving high accuracy.

### 5.1. Experimental Setup

**Figure 8:**
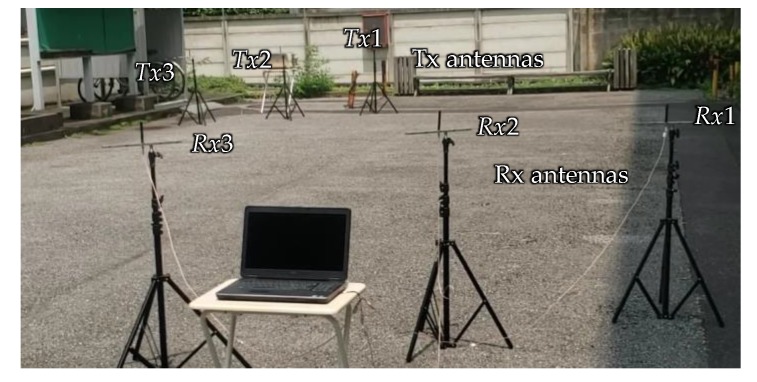
Photo of the experimental environment.

**Figure 9:**
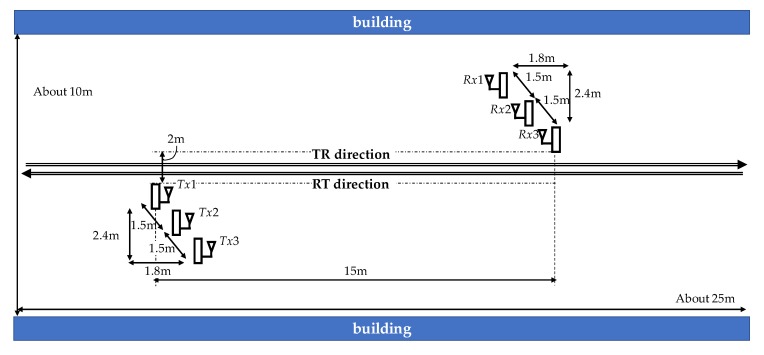
Antenna deployment.

We experimented on an outdoor road of our campus, as shown in Figure 8. The antennas’ deployment is shown in Figure 9. The size of the experimental area is about 10 × 25 m. The distance between the buildings on both sides is about 10 m. In the center of Tokyo, roads are narrow, and buildings are on both sides. Our experiment environment is similar to a road in the center of Tokyo. Note that the antenna cannot be placed diagonally in a real situation.

The number of transmitting and receiving antennas was three. The antenna height was set to 1 m, and the antenna spacing was set to 1.5 m in the transmitting and receiving antennas. When an antenna spacing is set as large, the passing section of human flow is extensive. Due to the limitations of the experimental area, we set the antenna spacing to 1.5 m. The reason why the antenna height was set to 1 m is as follows. The target object is human, and the average height of humans in Japan is about 160 cm for men and 150 cm for women, and about 170 cm for men and 160 cm for women in their 20s. The examinees in the experiment were in their 20s. If the antenna is close to the ground, the propagation is affected by ground reflections. To detect a human, it is necessary to utilize diffraction and scattering generated when the human body becomes an obstacle. Thus, the antenna height has to be set to less than 160 cm in the experiment. We set the antenna height to 1 m to create an environment in which diffraction and scattering always occur.

The transmitter and receiver operate the injection mode in Linux 802.11n CSI tool. The transmitter sends frames with a 1 ms interval using 5 GHz. We set the channel number to 100 ch. In this channel, we checked the channel state in advance, and the interference was the lowest among 5 GHz channels. IEEE802.11 wireless LAN has a carrier sense multiple access with collision avoidance (CSMA/CA) function to avoid frame collisions. That is, in the environment where the effect of interference is significant, the transmitter cannot send frames using this function. Since the CSI is obtained whenever the receiver receives frames in Linux 802.11n CSI Tool, there is no problem in the environment where the receiver can receive the frames.

The human activity of flow comprises seven types: one, two, and three people walking; one, two, and, three people running; and one person cycling. People walked or ran, keeping a certain distance from the person in front. As shown in Figure 8, we set the movement of flow in two directions: TR direction (transmitter side to receiver side) and RT direction (receiver side to transmitter side). The number of trials in each activity and each direction was 15. Note that, in our experiment, there were no people except examinees between transmitting and receiving antennas.

### 5.2. Experimental Results

Prediction performance was evaluated by leave-one-out cross-validation. We evaluated the prediction performance for each direction and two directions, TR and RT directions, as shown in Figure 9.

**Figure 10:**
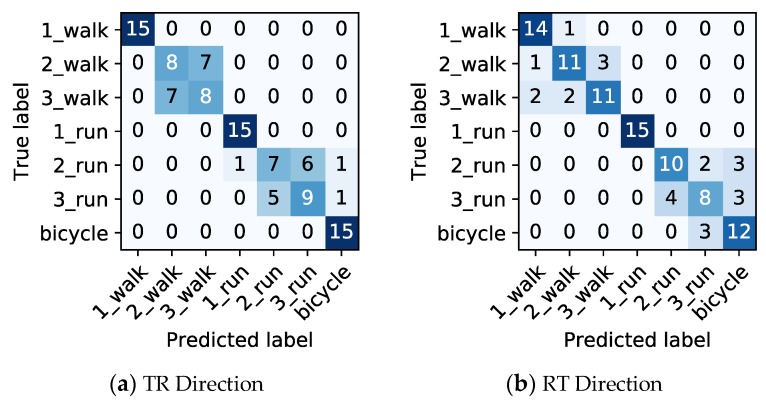
Confusion matrix for transmitter side to receiver side (TR) and receiver side to transmitter side (RT) directions when using only the lengths of the final passing section.

At first, we analyzed the performance when only the lengths of the final passing section were used. The prediction accuracies for TR and RT directions are 0.733 and 0.771, respectively. The definition of prediction accuracy is the ratio of the number of correct predictions and the total number of predictions. In the case of TR direction, the number of correct predictions was 77, and the total number of predictions was 15 × 7 = 105. Thus, the prediction accuracy is 77/105 = 0.733. In the case of RT direction, the number of correct predictions was 81, and the total number of predictions was 15 × 7 = 105. Thus, the prediction accuracy is 81/105 = 0.771. The confusion matrix for TR and RT directions is shown in Figure 10. For example, “1_walk” denotes one person walking. The prediction error occurs when the difference in the length of the final passing is small, such as two and three people walking. There is a similar trend between TR and RT directions.

Next, we analyzed the performance when only the CSI was used. The prediction accuracy is shown in Table 1. In this table, the “combination” denotes when $\alpha_{R_{x}T_{x},k}$ and $\gamma_{R_{x}T_{x},k}$ and/or $\beta_{R_{x}T_{x},k}$ and $\delta_{R_{x}T_{x},k}$ are used as feature values, and the “all of the above” denotes when the mean and variance of amplitude and phase are used as feature values. Compared with the accuracy using only the lengths of the final passing section, the prediction accuracy using only CSI increases in most cases of feature values used in Table 1. The feature values that achieve good performance are the mean and variance of amplitude and phase (row of “all of the above”) and the mean of amplitude, $\alpha_{R_{x}T_{x},k}$, and $\gamma_{R_{x}T_{x},k}$ (top row in the column of “combination”). In some of the feature values, there is a difference in the accuracy between TR and RT directions, but the feature values that achieve good accuracy are the same between them.

We analyze the performance when using the length of the final passing section and CSI. The prediction accuracy is shown in Table 2. Compared with Table 1, the accuracy increases in all cases by adding the lengths of the final passing section as the feature values. The common point between TR and RT directions in Table 2 is that the accuracy with 100% is achieved when the mean of amplitude, $\alpha_{R_{x}T_{x},k}$, and $\gamma_{R_{x}T_{x},k}$ (top row in the column of “combination”) are used. The accuracy is also good when the mean and variance of amplitude and phase (row of “all of the above”) are used. We consider the feature values in terms of computational complexity. The accuracy increases when the length of the final passing section and CSI compared with when only the length of the final passing section or only the CSI is used. Thus, the length of the final passing section and CSI is required to achieve good accuracy. The CSI amplitude is used to detect human flow and to derive the feature values, but the CSI phase is used only to derive the feature values. The CSI amplitude needs to be used to get the lengths of the final passing section. If only the CSI amplitude is used, the computational complexity is reduced compared with when the CSI amplitude and phase are used. Focusing on the feature values concerning CSI amplitude, the feature values that achieve the best accuracy are mean of amplitude, $\alpha_{R_{x}T_{x},k}$, and $\gamma_{R_{x}T_{x},k}$. In this case, even if the lengths of the final passing section as feature values are not used, good accuracy is achieved. In terms of the feature values to achieve good accuracy, the TR and RT directions are of a similar tendency, so the number of trials is sufficient for the evaluation.

Finally, we analyzed the performance for two directions (i.e., TR and RT directions), when using the length of the final passing section and CSI. The prediction accuracy is shown in Table 3. As with Table 2, the prediction accuracy is the best when the feature values are the mean of amplitude, $\alpha_{R_{x}T_{x},k}$, and $\gamma_{R_{x}T_{x},k}$. The number of prediction errors is only one; “three people running” was mistaken for “two people running”. Since the number of trials in each activity and each direction is 15 and the number of activities is 7, the total number is 15 × 7 × 2 = 210. Since the number of prediction errors is one, and the number of correct predictions is 209 (=210 − 1), the accuracy is 209/210 = 0.995. Based on this result, even if the training data of two directions is used, we can see that high prediction accuracy is achieved.

Summarizing the above, we clarified that the length of the final passing section and the CSI have to be used as feature values to achieve good accuracy. Considering the computational complexity, we also clarified that the feature values to achieve the best accuracy are mean of amplitude, $\alpha_{R_{x}T_{x},k}$, $\gamma_{R_{x}T_{x},k}$, and the passing time.

## 6. Discussion

We compare our system with the conventional method in terms of accuracy and the number of feature values. Reference [16] conducted classification by SVM as the conventional method. The feature values for the SVM are the normalized standard deviation of CSI (STD), the offset of signal strength (SS), the period of the motion (PM), the median absolute deviation (MAD), and interquartile range (IQR). These values are according to reference [13], which exploited Wi-Fi CSI for fall detection.

We applied these feature values to our system, and the prediction accuracy is shown in Table 4. Even if the conventional method is used, almost the same accuracy as our system is achieved. In terms of the number of feature values, there is a massive difference between them. The number of feature values to achieve the best accuracy in our system is 2 eigenvalues ($\alpha_{R_{x}T_{x},k}$ and $\gamma_{R_{x}T_{x},k}$) × 1 (mean) × 9 paths + 1 length of final passing section × 9 paths = 27. On the other hand, the number of feature values in the conventional method is 3 (STD, MAD, and IQR) × 30 subcarriers × 9 paths + 1 PM × 9 paths + 1 SS × 3 receiving antennas = 822. Therefore, our system achieves the best accuracy using a smaller number of feature values compared with the conventional method.

## 7. Conclusions

This paper proposes the human flow prediction method. To predict the activity of human flow, we used nine types of feature values derived from the CSI amplitude, the CSI phase, and the passing time across transmitting and receiving antennas. The CSI characteristics can express the effects of diffraction and scattering in the wireless signals, and the passing time depends on the length of human flow. At first, the proposed system detects that the human flow moves across transmitting and receiving antennas. From the passage detection, the passing time between antennas is obtained as the feature values. While the human flow is moving across between antennas, the CSI amplitude and phase fluctuate. In this passing section, the feature values are obtained from the mean and variance of the maximum eigenvalue in the auto-correlation matrix and variance–covariance matrix composed of the CSI amplitude and phase. In the experiment, we set seven types of activities: one, two, and three people walking; one, two, and three people with running; and one person cycling. The maximum prediction accuracy was 100% for each direction and 0.995 for two directions. The number of feature values to achieve the best accuracy in our system is decidedly smaller than that of the conventional method.

The contribution points of this paper are as follows. First, the antenna spacing in the transmitter and receiver is physically increased to extend the scanning area of CSI. The effect of the wide antenna spacing makes it easier to acquire exactly the activity and the passing time of the human flow. Second, the feature values to achieve the best accuracy are clarified. Considering the computational complexity, it is clarified that the feature values to achieve the best accuracy are the combination of the passing time and the mean of amplitude derived from the maximum eigenvalue of the auto-correlation matrix and the variance–covariance matrix composed of the CSI amplitude.

A challenging issue for future research is the examination of antenna setting methods. In this paper, the antenna spacing was set to 1.5 m, and the scanning area for CSI was extended. We are planning to evaluate the prediction accuracy using different antenna spacing. The optimum sampling frequency will also be discussed in our future research.

## Figures and Tables

**Table 1:** Prediction accuracy when using only the CSI (without the length of the final passing section). The values denote the predicted accuracy.

		Auto-Correlation Matrix	Variance–Covariance Matrix	Combination
		α(amplitude)/β(phase)	γ(amplitude)/δ(phase)	α,γ(amplitude)/β,δ(phase)
Feature values	(**a**) TR Direction.			
	mean of amplitude	0.914	0.648	0.952
	variance of amplitude	0.819	0.848	0.905
	mean of phase	0.867	0.857	0.876
	variance of phase	0.867	0.886	0.867
	mean and variance of amplitude	0.933	0.819	0.943
	mean and variance of phase	0.895	0.895	0.895
	all of the above	0.962	0.962	0.962
(**b**) RT Direction.				
Feature values	mean of amplitude	0.914	0.781	0.971
	variance of amplitude	0.705	0.686	0.867
	mean of phase	0.876	0.867	0.933
	variance of phase	0.829	0.771	0.819
	mean and variance of amplitude	0.876	0.876	0.971
	mean and variance of phase	0.876	0.876	0.905
	all of the above	0.971	0.971	0.981

**Table 2:** Prediction accuracy when using the length of the final passing section and CSI. The values denote the predicted accuracy.

		Auto-Correlation Matrix	Variance–Covariance Matrix	Combination
		α(amplitude)/β(phase)	γ(amplitude)/δ(phase)	α,γ(amplitude)/β,δ(phase)
(**a**) TR direction.				
Feature values	mean of amplitude	0.990	0.895	1.000
	variance of amplitude	0.990	0.895	0.981
	mean of phase	0.952	0.971	0.943
	variance of phase	0.933	0.933	0.914
	mean and variance of amplitude	0.962	0.962	0.971
	mean and variance of phase	0.952	0.933	0.943
	all of the above	0.981	0.971	0.971
(**b**) RT direction.				
Feature values	mean of amplitude	0.990	0.981	1.000
	variance of amplitude	0.990	0.981	0.952
	mean of phase	0.962	0.971	0.971
	variance of phase	0.981	1.000	0.981
	mean and variance of amplitude	0.981	0.962	0.990
	mean and variance of phase	0.990	1.000	0.990
	all of the above	1.000	1.000	1.000

**Table 3:** Prediction accuracy for two directions when using the length of the final passing section and CSI. The values denote the predicted accuracy.

		Auto-Correlation Matrix	Variance–Covariance Matrix	Combination
		α(amplitude)/β(phase)	γ(amplitude)/δ(phase)	α,γ(amplitude)/β,δ(phase)
Feature values	mean of amplitude	0.986	0.905	0.995
	variance of amplitude	0.986	0.905	0.986
	mean of phase	0.938	0.957	0.952
	variance of phase	0.952	0.962	0.943
	mean and variance of amplitude	0.971	0.933	0.990
	mean and variance of phase	0.976	0.971	0.971
	all of the above	0.995	0.990	0.990

**Table 4:** Prediction accuracy in the conventional method used in [16].

TR-Direction	RT-Direction	Two Directions
1.000	0.990	0.995

## Appendix A

One of the objectives of the paper is to clarify the feature values to achieve the best accuracy, not to compare machine learning algorithms. The appendix is the evaluation in the case of random forest. Table A1 shows the prediction accuracy using random forest. Compared with Table 3, in terms of accuracy, the better or worse method depends on the feature values. However, the feature values to achieve better accuracy are the same, the mean of amplitude, $\alpha_{R_{x}T_{x},k}$, and $\gamma_{R_{x}T_{x},k}$ (top row in the column of “combination”), and the mean and variance of amplitude and phase (row of “all of the above”). Considering the computational complexity, the feature values to achieve the best accuracy are the mean of amplitude, $\alpha_{R_{x}T_{x},k}$, and $\gamma_{R_{x}T_{x},k}$.

The reason for selecting SVM is based on [22,23,24]. In these references, the accuracy of using SVM outperforms using random forest. Reference [24] described that the linear SVM indicates the best performance on a small dataset. Since the size of the dataset is small in our study, we selected linear SVM.

**Table A1:** Prediction accuracy for two directions when using the length of the final passing section and CSI. The machine learning algorithm is random forest. The values denote the predicted accuracy.

		Auto-Correlation Matrix	Variance–Covariance Matrix	Combination
		α(amplitude)/β(phase)	γ(amplitude)/δ(phase)	α,γ(amplitude)/β,δ(phase)
Feature values	mean of amplitude	0.962	0.929	0.981
	variance of amplitude	0.962	0.929	0.967
	mean of phase	0.929	0.943	0.948
	variance of phase	0.952	0.943	0.957
	mean and variance of amplitude	0.971	0.943	0.971
	mean and variance of phase	0.962	0.962	0.971
	all of the above	1.000	0.976	1.000
